# The Glasgow Hospital for Sick Children

**Published:** 1888-03-03

**Authors:** 


### THE GLASGOW HOSPITAL FOR SICK CHILDREN.

An esteemed correspondent sends us the following interesting and instructive sketch :—"The Glasgow Hospital for Sick Children, which is now in the sixth year of its existence has, owing to the success attending it, become one of the most valuable institutions of the city. Very important and successful work has been accomplished, and operations of exceptional importance performed.

Situated in close proximity to a densely-populated district chiefly of the poorer class, its benefits have been largely taken advantage of, not only by them, but by others from all parts of the city and the surrounding counties.

As the hospital became more generally known the applications for admission so increased, that the accommodation of 58 beds was found insufficient; many applicants had to be refused, and with regret the directors found themselves unable to meet the increasing demand.

Fortunately, a house adjoining the hospital came into the market, and was purchased; and through the generosity of Mr. Thomas Carlile, chairman of directors, a portion of it was altered and converted into an additional ward for 12 medical beds, which has been quite full since its opening in October last. The cost of the alterations paid by Mr. Carlile amounts to £1,100, and, in addition, he has agreed to contribute £200 for three years towards the maintenance of the ward. The directors have named the ward, "The Carlile Ward," in recognition of Mr. Carlile's gifts and his kind interest in the hospital.

The Hospital has now 70 beds, of which 32 are medical and 38 surgical.

From the opening of the hospital until December 31st last, 1,850 patients had been treated, and it is gratifying to notice that of these 1,108 have been discharged as cured, 393 improved, 191 unimproved or taken out before any benefit derived, while there were 158 deaths. Many of the patients, however, were in a dying state before being received. During the same period 564 operations have been performed, many of them of special importance. The following figures are interesting.

| | 1884. | | | 1885. | | | 1886. | | | 1887. | | |
|---|---|---|---|---|---|---|---|---|---|---|---|---|
| Average | £ | s. | d. | £ | s. | d. | £ | s. | d. | £ | s. | d. |
| Daily expenditure | 5 | 16 | 5·5 | 5 | 16 | 3·4 | 5 | 18 | 3·1 | 6 | 7 | 2 |
| Daily cost of each patient........ | 0 | 2 | 5·4 | 0 | 2 | 3·5 | 0 | 2 | 3·8 | 0 | 2 | 2·8 |
| Total cost of each patient treated | 4 | 13 | 1·2 | 4 | 13 | 2·4 | 4 | 6 | 0·8 | 4 | 19 | 6 |
| Yearly cost of each cot ...... | 44 | 14 | 3 | 41 | 16 | 5·5 | 42 | 6 | 5·9 | 40 | 15 | 9·3 |

In connexion with the hospital a dispensary is being erected from plans by Messrs. Campbell, Douglas, and Sellars. It will prove a valuable adjunct.

The hospital is to a limited extent endowed, the result of a fancy fair organised by several influential ladies, but otherwise it is wholly dependent on voluntary contributions, and it is not yet self-supporting. The Ladies' Auxiliary Association give a very valuable assistance, nearly one-half of the subscriptions being received through their aid. The deficiency in the ordinary income for the year ending December 31st last was £280 18*s.* 11*d.* The useful work done by the hospital is so manifest that strenuous efforts should be made to prevent its effieiency being impaired by a too limited income."

